# A Comprehensive Evaluation of Primary Breast Augmentation Education Videos for Surgical Trainees

**DOI:** 10.1093/asjof/ojaf018.014

**Published:** 2025-05-13

**Authors:** Lauren Powell, John Miskella, Lily Qian, George Landis, Minh-Doan Nguyen, Jennifer Harrington

**Affiliations:** University of Minnesota, Minneapolis, MN; University of Minnesota, Minneapolis, MN; University of Minnesota, Minneapolis, MN; University of Minnesota, Minneapolis, MN; University of Minnesota, Minneapolis, MN; Private Practice, Plymouth, MN

## Abstract

**Goals/Purpose:**

Aesthetic breast augmentation surgeries were the second most common surgical aesthetic procedure in the US in 2023.1 In a survey of residents and 4th-year medical students, 95% reported using online videos as a tool to learn about surgical procedures, the majority citing YouTube as the most common source.2 While videos can be a crucial learning tool for trainees, it is important that these resources undergo a thorough evaluation to establish their educational value. While prior studies in the otolaryngology and general surgery literature have investigated the quality of surgical YouTube videos,3,4 no studies have examined the quality of videos related to breast augmentation. Thus, the objective of this study is to compare the quality of breast augmentation educational videos.

**Methods/Technique:**

A total of 640 intraoperative primary breast augmentation YouTube videos and 3,527 videos published in the *Journal of Plastic and Reconstructive Surgery (PRS)* video gallery were screened using predefined search terms. With the help of 3 attending surgeons who routinely perform breast augmentation, a 14-point rubric was created to identify critical perioperative and intraoperative surgical criteria that should be included in educational videos regarding primary breast augmentation. Each video was also assessed on audiovisual quality. All included videos were graded on a binary scale by two independent reviewers and interrater reliability was assessed using Kappa’s coefficient. Statistical significance was defined as a *P* < 0.05.

**Results/Complications:**

A total of 11 Youtube videos, and 7 *PRS* videos met inclusion criteria. All *PRS* videos were deemed high quality, while only 45% of YouTube videos were rated as high quality. Moreover, the *PRS* videos were of a statistically significant higher quality than the YouTube videos, with an average score of 11.1 out of the 14 criteria compared to 9.0 respectively (*P* = 0.03). The videos posted to the academic video gallery were rated as higher quality in all three categories; audiovisual, perioperative and intraoperative criteria. The number of views, likes, duration, time since upload or channel subscribers were not strong predictors of the quality of YouTube videos. A high degree of interrater reliability was confirmed using Kappa's coefficient, with κ = 0.97.

**Conclusion:**

This study found that the quality of surgical education videos on breast augmentation greatly varied from video to video within both Youtube and the *PRS* website. Videos within the peer-reviewed journal scored higher than those on Youtube. Trainees in prior studies have cited Youtube as a major source for educational content, and thus, are especially vulnerable to this low quality, as they may not be able to differentiate between good quality and inadequate content. Additionally, there was not a strong correlation with any of the commonly used metrics such as views and likes that a trainee might use to help identify higher quality content on Youtube. Thus, surgical trainees should use caution when deciding how to supplement their learning with online videos. A more reliable option for high quality surgical content is to watch videos posted to a peer-reviewed academic journal. Even with this, setting standards for video quality and essential criteria is useful for creating educational content through video content.
